# Demographic and Clinico-Epidemiological Features of Dengue Fever in Faisalabad, Pakistan

**DOI:** 10.1371/journal.pone.0089868

**Published:** 2014-03-03

**Authors:** Faiz Ahmed Raza, Shafiq ur Rehman, Ruqyya Khalid, Jameel Ahmad, Sajjad Ashraf, Mazhar Iqbal, Shahida Hasnain

**Affiliations:** 1 Pakistan Medical Research Council, Research Centre, Punjab Medical College, Faisalabad, Pakistan; 2 Department of Microbiology and Molecular Genetics, University of the Punjab, Quaid-e-Azam Campus, Lahore, Pakistan; 3 School of Biological Sciences, University of the Punjab, Quaid-e-Azam Campus, Lahore, Pakistan; 4 Department of Pathology, Allied Hospital, Faisalabad, Pakistan; 5 Nano Bio Energy Engineering School of Integrative Engineering, Chung-Ang University, Seoul, Korea; University of Texas at El Paso, United States of America

## Abstract

This cross-sectional study was carried out to explore the epidemiological and clinical features of dengue fever in Faisalabad, Pakistan during 2011 and 2012. During the study period, anti-dengue IgM positive cases were reported in the post-monsoon period during the months of August–December. Certain hotspots for the dengue infection were identified in the city that coincide with the clusters of densely populated urban regions of the city. Out of total 299 IgM positive patients (male 218 and female 81); there were 239 dengue fever (DF) and 60 dengue hemorrhagic fever (DHF) patients. There was decrease in the median age of dengue patients from 31 years in 2011 to 21.5 years in 2012 (p<0.001). Abdominal pain was seen in 35% DHF patients followed by nausea in 28.3%, epistaxis in 25% and rash in 20% patients (p<0.05). Patients reported to be suffering from high-grade fever for an average of 8.83 days in DHF as compared to 5.82 days in DF before being hospitalized. Co-morbidities were found to be risk factor for the development of DHF in dengue patients. Clinical and laboratory features of dengue cases studied could be used for the early identification of patients at risk of severe dengue fever.

## Introduction

Dengue is an arthropod-borne arboviral disease becoming major public health concern, both in the tropical and subtropical regions of the world. It is estimated that 50 to 100 million cases of dengue virus (DENV) infection reported annually; which leads to 250,000 to 500,000 cases of dengue hemorrhagic fever (DHF) or dengue shock syndrome (DSS) and 20,000–25,000 deaths. The causative agent belongs to family Flaviviridae; currently there are 4 distinct serotypes: DENV-1, DENV-2, DENV-3 and DENV-4; being transmitted to humans principally by *Aedes aegypti* and *Aedes albopictus* mosquitoes. Dengue infection results in a wide spectrum of clinical symptoms ranging from mild dengue fever (DF) to the very severe form of the disease, DHF and DSS [1], [2].

Pakistan, the sixth most populated country (185 million people) of the world located in south Asia between latitudes 23.45° and 36.75° north and longitudes 61° and 75.5° east. Due to its subtropical location and suitable climatic conditions for vectors, it has been source of many vector born diseases including malaria, leishmaniasis, Crimean Congo hemorrhagic fever, dengue hemorrhagic fever, West Nile virus infection, Japanese encephalitis and scrub typhus [3], [4]. Although dengue virus exists in this region even before creation of Pakistan [5], the first outbreak of DF due to DENV-1 and DENV-2 was reported in 1994 leading to morbidity in thousands [6]. Decade after first outbreak, a second outbreak involving DENV-3 occurred in 2005 in Karachi with a sudden increase in number of DHF patients. The increase in disease severity might be contributed by infection with DENV-3, in a population sensitized previously with DENV-1 and DENV-2, through antibody dependent enhancement (ADE) [7]. Afterwards, another outbreak occurred in 2006 affecting the population in the north to south of Pakistan leading to 3500 confirmed cases and 52 deaths. Analysis of the selected samples revealed co-circulation of DENV-2 and DENV-3 in the population [8]. During 2010 and 2011, Pakistan was hit by devastating floods that had not only destroyed property and lives but also provided breeding sites for dengue virus vectors, resulting in worst outbreaks during these years [6].

Various small scale and large scale studies had been carried out previously in various cities of Pakistan [7]–[9]. However, there is severe deficiency of thorough studies related to dengue outbreaks in Faisalabad. In this direction, we present comprehensive dengue infection epidemiology in the Faisalabad city during 2011–2012. The patient's data was used to identify possible hotspots for the dengue infection in the city during the two years of study. The possible effect of prevailing weather conditions, at the time of epidemic, on the frequency of dengue cases was investigated. In addition, patient's demographic and clinical features were studied that can facilitate in the early identification of patients suffering from severe form of dengue fever.

## Materials and Methods

### Ethical Clearance

Ethical clearance was taken from institutional ethical review committee of Punjab Medical College, Faisalabad before starting the study. Written informed consent was taken from all patients or legal guardians.

### Study Setting and Patient Data

Faisalabad, the second largest city in the province of Punjab and third largest city of Pakistan, located between latitudes 31.41°N and longitude 73.11°E, having a population of more than 2.6 million [10], is considered as an industrial hub of Pakistan. Two major public sector hospitals in the city are Allied Hospital (1150 beds) and DHQ Hospital (600 beds). This cross-sectional study was carried out in 2011 and 2012. All clinically confirmed cases of dengue fever who were admitted to the high dependency unit of Allied Hospital and DHQ Hospital Faisalabad for its management were included in the study. Diagnosis of dengue was confirmed if apart from high grade fever these patients also had positive anti-dengue IgM antibodies. All patients were interviewed for signs and symptoms related to dengue fever and they were followed daily till their discharge from the hospital for any change in their clinical signs and laboratory parameters. The data for confirmed cases of dengue included their demographics, co-morbidity, previous exposure, length of hospital stay, hemorrhagic manifestations and clinical signs and symptoms.

Dengue cases were classified into Dengue Fever (DF) and Dengue Hemorrhagic Fever (DHF) according to World Health Organization (WHO) classification and case definition [11]. A patient was considered as a confirmed case of DF if presented with fever of 2–7 days duration and having two or more of the following symptoms: headache, retro-orbital pain, myalgia/arthralgia, rash, hemorrhagic manifestations (petechiae and positive tourniquet test) and, leukopenia. The patients of DHF were distinguished from DF if they had (1) fever for 2–7 days; (2) one or more hemorrhagic manifestations (positive tourniquet test, petechiae, ecchymosis or purpura, bleeding from mucosa, injection sites or other sites); (3) Thrombocytopenia (platelet count <100,000/mm^3^); and (4) evidence of plasma leakage. A patient was confirmed to have dengue virus infection if IgM anti-dengue antibodies were detected in the patient's serum after 5 days of fever. Dengue IgM antibodies were measured by indirect IgM enzyme linked immunosorbant assay (ELISA), using commercially available kit (Human GmbH, Wiesbaden, Germany). Antibody index of >0.496 was considered as positive for dengue virus infection. Patients having an index value less than the cutoff and reported with other febrile illness were excluded from the study. Furthermore, data for the patients having co-morbidities is discussed separately to the data for the patients with dengue infection alone.

### Diagnostic Laboratory Values

Platelet count less than 150,000 cells/mm^3^ blood was defined as thrombocytopenia. Leukocyte count of less than 4.5 cells/mm^3^ was defined as leucopenia. Hematocrit (HCT) of >48% was considered as elevated. Hemoglobin (Hb) concentration less than 13 and 12 g/L was considered as decreased in males and females respectively while in children values less than 11.2 g/L was considered as low. The values of alanine aminotransferase (ALT) and aspartate aminotransferase (AST) above 45 U/ml and 36 U/ml respectively were considered as elevated.

### Cluster Map for Dengue Infection

Google Earth™ was used to build map of the Faisalabad city in which polygons represent residential units (towns, colonies etc.) whereas placemarks indicate areas from which dengue cases were reported. Number on the placemarks represent frequency of dengue cases from the specified area. Boundaries of individual residential units were determined in Google Maps™.

### Statistical Analysis

Statistical Package for Social Sciences (SPSS) (Chicago, IL), version 19 was used for data entry, processing and statistical analysis. p value was calculated by Wilcoxon test for age, temperature, hematocrit and fluid therapy, while χ^2^ test was used for all other categorical variables. A 2-tailed p value≤0.05 was considered to be statistically significant.

## Results

This two years cross-sectional study was carried out in 2011 and 2012. A total of 393 confirmed dengue patients, including 353 in 2011 and 40 in 2012, were enrolled in the study. Amongst them 94 (23.9%) cases of dengue fever were reported with single/multiple co-morbidities, which are discussed separately.

### Patient Characteristics

**Figure 1:**
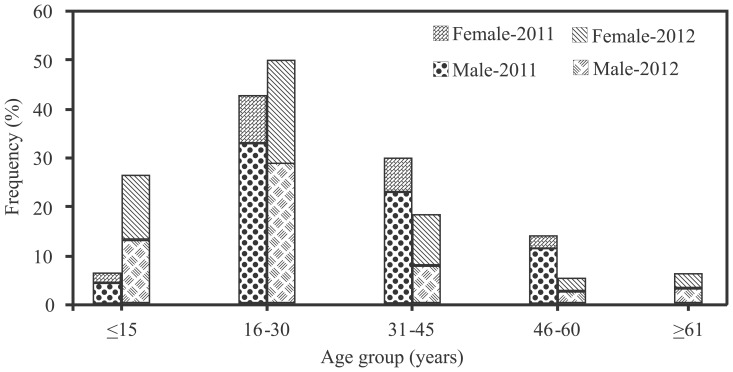
Gender wise distribution of dengue patients according to different age groups.

Out of 299 patients, 218 (72.9%) were males and 81 (27.1%) were females. The median age of the patients was 30 years and age ranged from 6 to 90 years, including 27 (9%) children (till 16 years) and 272 (91%) adults. However, no child was reported under the age of 6, during both years. There were 132 (48.5%) young adults (17 to 30 years old) among adult patients. There was decrease in the median age of patients from 31 years in 2011 to 21.5 years in 2012 (p<0.001). The highest prevalence of dengue patients was observed between 16 to 30 years (Figure 1). Table 1 gives yearly distribution of dengue cases according to different age groups and disease severity. Within age group of 6–15 years there was 20% increase in the prevalence of dengue patients, while there was 23.9% increase in DHF within age group of 6 to 30 years, in 2012 than the previous year. Table 2 presents demographic features, clinical characteristics and laboratory findings of dengue patients according to severity of the disease. DF was diagnosed in 239 patients (184 males, 55 females) and DHF in 60 patients (34 males, 26 females), with highest incidence (p = 0.03) of infection in male population. The median age of DF patients was 30 years and 24.5 years in DHF patients. DHF was reported more commonly in younger population (p = 0.05).

**Table 1:** Year Wise Distribution of Patients Suffering From Dengue Fever (DF) and Dengue Hemorrhagic Fever (DHF) According to Different Age Groups.

Age group (years)	Year-2011		Year-2012	
	DF (%)	DHF (%)	DF (%)	DHF (%)
≤15	9 (3.3)	5 (1.8)	3 (10.7)	6 (21.4)
16 to 30	98 (36.2)	23 (8.5)	10 (35.7)	5 (17.9)
31 to 45	67 (24.7)	13(4.8)	3 (10.7)	1 (3.6)
46 to 60	34 (12.5)	5 (1.8)	0.0	0.0
>60	15 (5.5)	2 (0.7)	0.0	0.0

**Table 2:** Characteristics Presented by Patients Suffering from Dengue Fever (DF) and Dengue Hemorrhagic Fever (DHF) Patients.

Characteristics	DF (%)	DHF (%)	N	p value^1^	
**Demographics**					
Age [median (interquartile range)]	30 (22)	24.5 (17)	299	0.05	
Gender					
Male	184 (77)	34 (56.7)	299	0.03	
Female	55 (23)	26 (43.3)			
**Clinical Presentation**					
Headache	184 (77)	46 (76.7)	299	>0.05	
Myalgia/arthralgia	212 (88.7)	49 (81.6)	299	0.191	
Vomiting	121 (50.6)	35 (58.3)	299	0.314	
Abdominal pain	34 (14.2)	21 (35)	299	0.001	
Nausea		36 (15.1)	17 (28.3)	299	0.023
Rash	16 (6.7)	12 (20)	299	0.005	
Diarrhea	11 (4.6)	5 (8.3)	299	0.331	
Anorexia	13 (5.4)	9 (15)	299	0.22	
Sore throat	16 (6.7)	6 (10)	299	0.407	
Retro-orbital pain	17 (7.1)	9 (15)	299	0.07	
Cough	22 (9.2)	7 (11.6)	299	0.625	
Cold skin/clammy skin	4 (1.7)	5 (8.3)	299	0.018	
Restlessness	11 (4.6)	7 (11.6)	299	0.062	
Periorbital puffiness	5 (2.1)	5 (8.3)	299	0.031	
**Hemorrhages**					
Epistaxis (Nose bleed)	14 (5.9)	15 (25)	299	<0.001	
Gingivitis (Gum Bleed)	17 (7.1)	11 (18.3)	299	0.012	
Melena (blood in stool)	9 (3.8)	12 (20)	299	<0.001	
Hematemesis (vomiting blood)	13 (5.4)	8 (13.3)	299	0.045	
Hematuria (blood in urine)	5 (2.1)	5 (8.3)	299	0.031	
Petechiae (small spots)	12 (5)	7 (11.7)	299	0.074	
Other*	8 (3)	7 (11.7)	299	0.016	
**Clinical Signs**					
Temperature [median (SD)]	37 (0.2)	37.22 (1.1)		0.108	
Abdominal tenderness	7 (2.9)	8 (13.3)	299	0.003	
Splenomegaly (Spleen enlargement)	15 (6.3)	14 (23.3)	299	<0.001	
Hepatomegaly (Liver enlargement)	12 (5)	18 (30)	299	<0.001	
**Laboratory Findings**					
Thrombocytopenia at presentation	226 (96.2)	57(95)	295	0.715	
Low hemoglobin	104 (44.1)	43 (71.7)	296	<0.001	
Hematocrit level on admission [median (SD)]	42.05 (8.3)	36 (17.9)	296	<0.001	
Leukopenia	172 (72.3)	42 (70)	298	0.749	
Increased ALT	112/213 (52.6)	23/31 (74.2)	244	0.032	
Increased AST	08/14 (57.1)	05/05 (100)	19	0.077	

1 p-value was calculated by non-parametric (Mann-Whitney U) test for age, temperature, and hematocrit. The χ^2^-test was used for all other categorical variables; p value less than 0.05 was considered as significant.

*Menstrual bleed, Sub-conjunctival bleed, Ear Bleed, Hemoptysis, Purpura, Ecchymosis.

### Clinical Signs and Symptoms

Myalgia was present in 87.3% patients, followed by headache (76.9%), vomiting (52.2%), abdominal pain (18.4%), nausea (17.7%), cough (9.7%), rash (9.4%), retro-orbital pain (8.7%), sore throat (7.4%), anorexia (7.4%), restlessness (6%), diarrhea (5.4%), periorbital puffiness (3.3%) and cold/clammy skin (3%) patients. Out of 299 patients, 143 (47.8%) patients presented with various types of hemorrhage, which included epistaxis in 9.7% patients, followed by gingivitis (9.4%), melena (7%), hematemesis (7%), petechiae (6.4%) and hematuria (3.3%) patients. Spleen and liver were enlarged in 29 (9.7%) and 30 (10%) patients, respectively.

Although, headache, myalgia/arthalgia and vomiting were the most common symptoms observed but they were not significantly different amongst DF and DHF patients. Conversely, abdominal pain, nausea, rash, cold/clammy skin and periorbital puffiness were significantly (p<0.05) different in DHF. Retro-orbital pain and restlessness were mostly, but not significantly, associated with DHF. Furthermore, spontaneous hemorrhages, abdominal tenderness, splenomegaly and hepatomegaly were most frequently (p≤0.05) observed in DHF (Table 2).

### Laboratory Findings

Liver involvement, as indicated by abnormal levels of liver enzymes, was observed in various dengue patients. Alanine aminotransferase (ALT) was raised 55.3%, whereas aspartate aminotransferase (AST) was raised in 68.4% patients. Increase in the levels of liver enzymes (AST and ALT) was most frequently associated with DHF. ALT levels were raised in 74.2% (p = 0.032), while AST levels were raised in all DHF patients (p = 0.077). ALT was 2.7 times higher, while AST was 10 times higher than the normal values in DHF patients. Majority of the patients (96%) presented with thrombocytopenia at time of admission. Low hemoglobin and leucopenia were observed in 49.6% and 71.8% of all patients, respectively. Median hematocrit level for all dengue patients was 41.3. Amazingly the hematocrit, an indicator of capillary leakage, was higher in DF patients (42%) in contrast to DHF patients (36%; p<0.001), at the time of hospitalization.

### Dengue Case Management

Table 3 presents data related to impact of severity of the disease on length of hospitalization, course of illness and management of dengue patients. Proper diagnosis and treatment of dengue patients is a primary clinical goal that could contribute significantly in the reduction of length of hospitalization and treatment costs but could also affect the outcome of the disease. DF patients were reported with high grade fever from average 6.7 days before being hospitalized. Mean length of hospital stay for DF was 3.8 days. However, severe form of the disease (DHF) has contributed substantially towards longer course of illness (8.83 fever days) and length of stay (4.9 days). Fluid replacement therapy was administered in case of hypotension, while whole blood and/or platelet concentrates were infused in those having severe thrombocytopenia, low hematocrit and capillary leakage. Furthermore, total 12 deaths were recorded (case fatality rate 2.5%) due to severe form of dengue fever (DHF/DSS), in 2011 only. The victims were suffering from multiple co-morbidities and were mostly died due to multiple organ failure.

**Table 3:** Impact of Disease Severity on Length of Hospitalization, Course of Illness and Management of Dengue Patients [mean (SD)].

Characteristics	DF	DHF	N	p value^1^
Days of fever (at presentation)	5.82 (2.7)	8.83 (6.2)	299	<0.001
Length of stay (days)	3.56 (1.4)	4.90 (2.5)	299	<0.001
Fluid administered (liters)	3.36 (2.1)	5.45 (5)	253	0.001
Number of blood transfusions	1.3 (1)	2.2 (2.1)	78	0.073
Number of platelet transfusions	1.23 (0.4)	1.5 (1)	54	0.494

1 p value was calculated by non-parametric (Mann-Whitney U) test; p value less than 0.05 was considered as significant.

### Co-morbidities and Outcome of Dengue Infection

Table 4 shows the frequency of various co-morbidities in dengue infection. Total 94 (23.9%) cases of dengue fever were reported with single or multiple co-morbidities, including 70 (74.5%) males and 22 (25.5%) females. Their median age was 33.4 years, including 16 (17%) children (till 16 years) and 78 (83%) adults. Attack rates with multiple co-morbidities increased constantly with the age and was highest among the age group of 46 to 50 years. Low hemoglobin and thrombocytopenia at time of admission were observed in 41 (87%) and 94 (100%) dengue patients, respectively. Splenomegaly, hepatomegaly and severe thrombocytopenia (platelet count ≤15,000 cells/mm^3^) were most frequently observed (p<0.05) in patients with co-morbidities as compared to DF patients. Low hemoglobin was observed most commonly, although not significantly (p = 0.089), in patients with co-morbidities than with DF. Hypotension, severe thrombocytopenia, low hematocrit and capillary leakage in patients with co-morbidities were managed with average 4.39 liters of intravenous fluids, 1.4±0.5 whole blood transfusions and 2±1.2 platelets transfusions. Fluid replacement therapy, number of whole blood and platelets transfusions were significantly higher (p<0.05) in patients with co-morbidities than in DF patients. DF was diagnosed in 62 (49 males, 13 females) and DHF in 32 (21 males, 11 females) patients.

**Table 4:** Frequency of Different Co-morbidities in Dengue Patients.

	A	B	C	D	E	F	G	H	I	J	K
**A**	48										
**B**	1	1									
**C**	0	0	1								
**D**	1	0	0	7							
**E**	1	1	0	1	9						
**F**	0	0	0	0	0	2					
**G**	0	0	0	0	0	4	3				
**H**	0	0	0	0	0	2	1	0			
**I**	0	0	0	0	0	0	1	0	0		
**J**	0	0	0	0	0	0	0	0	0	1	
**K**	0	0	0	0	0	0	0	0	0	0	1
**L**	1	0	0	0	0	0	0	0	0	0	0
**M**	1	0	0	0	0	0	0	1	0	0	0

**A**, Hepatitis C; **B**, Hepatitis B; **C**, Hepatitis A; **D**, Typhoid; **E**, Malaria; **F**, Diabetes; **G**, Hypertension; **H**, Ischemic heart disease; **I**, Urinary tract infection; **J**, Cancer; **K**, Hepatitis C, Malaria, Typhoid, Abdominal tuberculosis, Diabetes, Hypertension and Ischemic heart disease; **L**, Hepatitis B and Malaria; **M**, Diabetes and Hypertension.

Among the co-morbidities 53 (56.4%) of the dengue patients were co-infected with hepatitis C virus (HCV), including one with diabetes mellitus, hypertension, typhoid, ischemic heart disease and abdominal tuberculosis; one with hepatitis B virus co-infection; one with typhoid and another one with malarial parasite. Data for the 49 dengue patients with HCV co-infection was compared exclusively with the patients with dengue infection alone. No significant (p>0.05) difference was observed in the clinical signs and symptoms in the dengue patients and those with the HCV co-infection. However, hemorrhagic tendencies were significantly higher (p = 0.02) in patients with HCV co-infection. DF was diagnosed in 36 (73.5%) and DHF in 17 (26.5%) HCV co-infected dengue patients.

Total 12 (9 males, 3 females) patients were reported with *Plasmodium vivex* co-infection, among them one was also co-infected with HCV and HBV; one with HBV alone and one with *Salmonella typhae* alone. 50% of the dengue patients with malarial parasite (MP) co-infection developed DHF. Among the three female patients having malarial co-infection two were pregnant (38 weeks pregnancy). Both of them were diagnosed as cases of DHF with severe internal bleeding (HCT<29%), various hemorrhages, ulcers on the lips and anemia, resulting in intrauterine death of the fetuses.

### Clusters of Dengue Infection in the City

**Figure 2:**
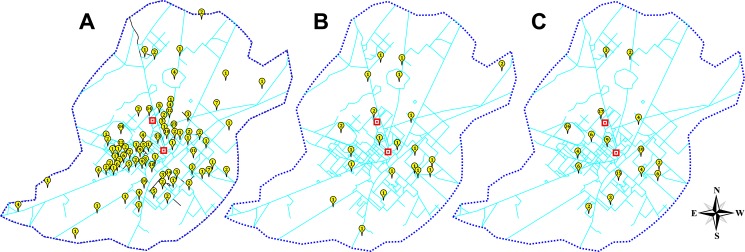
Hot spots for dengue infection in Faisalabad city. The distribution of dengue cases reported from Faisalabad city. A) Map of Faisalabad city showing areas from which dengue cases were reported in 2011, B) in 2012 and during both years. Squared place marks represents location of medical centers in the city.

Figure 2 shows possible hot spots in Faisalabad city from which dengue infections were reported more frequently. The patients were reported from 14 adjoining and 3 distant districts of province Punjab, including the major share from Faisalabad city alone, followed by other neighboring districts. The cases reported from Faisalabad city were from both of its less populated peripheral regions, as well as, from its densely populated central regions. Comparing the data for both years indicate high incidence of infection from various overlapping regions in the Faisalabad city. Furthermore, these hot spots coincide with the clusters of densely populated central regions. More recently, infections from new areas adjacent to these hot spots reported around the city.

### Environmental Factors and Frequency of Dengue Cases

**Figure 3:**
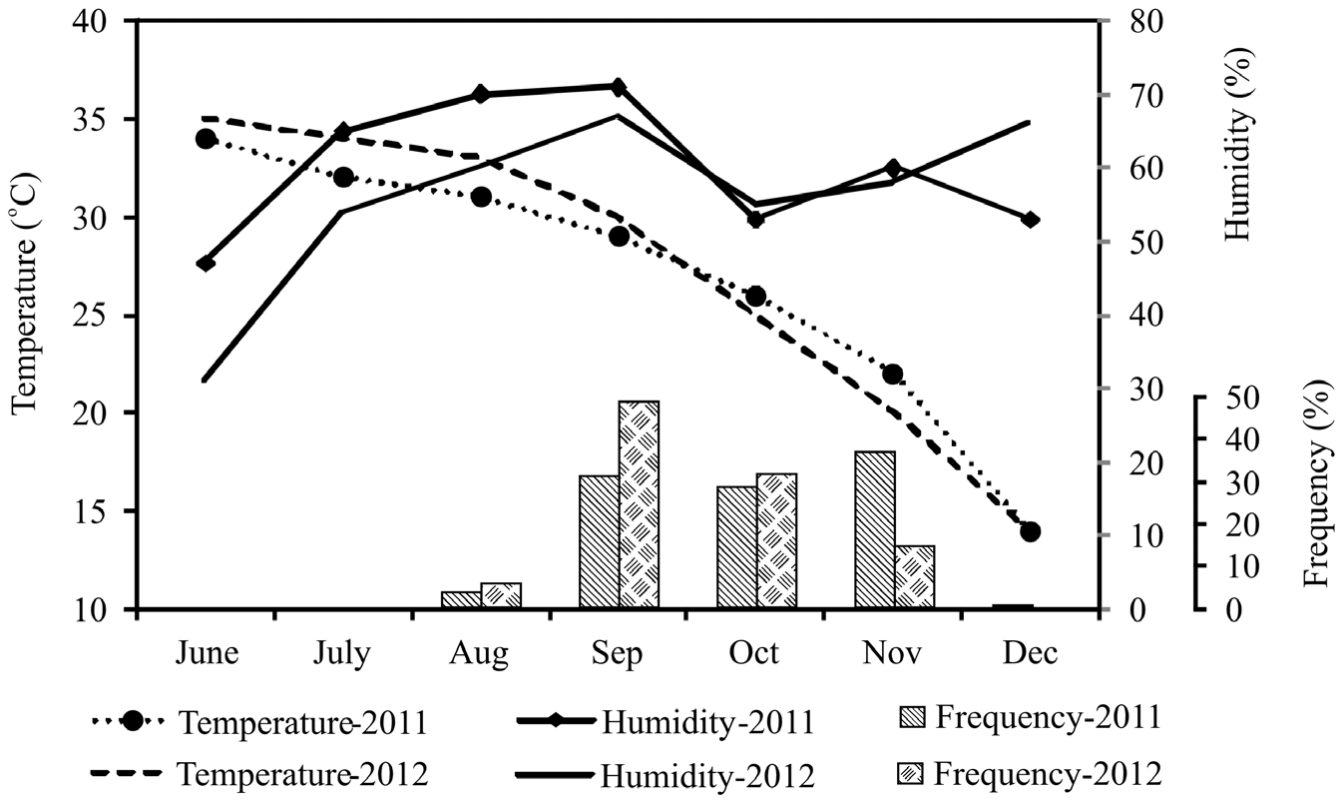
Effect of humidity and temperature on the frequency of dengue cases. Solid and dotted lines represents humidity and temperature respectively, whereas vertical bars represents frequency of dengue cases during the months of July to December.

Figure 3 gives monthly average humidity, temperture and frequency of dengue patients reported during the study period. The onset of dengue virus epidemics during the study period coincide with the post-monsoon era, starting from August, reaching its peak in September/October and declining in November/December, both years. The highest and the lowest monthly temperature was recorded during June (35°C), and December (14°C) respectively in both years. Similarly, the highest and the lowest monthly relative humidity (RH) was recorded during September (71%) and June (31%) respectively. In contrast to subsequent year, second peak of dengue virus infection was observed in November-2011 with highest prevalence (36.7%) in that year. Mid of 2012 was warmer (average temperature during June–Sep was 33°C) and dryer (average RH during June–September was 53%) than the previous year (average temperature 31.5°C; RH 63.3%), accompanied by rapid decline in temperature by the end of year.

## Discussion

The dengue virus has threatened half of the world's population, hence becoming a major public health problem especially in tropical countries [12]. The central place of Faisalabad city amongst surrounding districts having underdeveloped health settings makes it a hotspot for health care seekers. Dengue patients were reported from the densely populated central regions of the Faisalabad city. Certain hotspots were also detected from which dengue cases were reported more frequently both years. In addition, new cases of dengue fever from adjacent regions of these hot spots show the spread of dengue infection to adjacent areas. Both year data also indicate outwards progression of dengue infection from the populated central areas to adjoining less-populated regions of the city.

The dynamics of the dengue fever epidemic are influenced by the complex interactions among virus, vector, host and directly dependent on climatic and environmental conditions [13]. The peak incidence of dengue virus infection was observed from September to November during both years of study. Seasonal humidity and temperature variations have role in vector survival that ultimately linked with emergence of dengue epidemics. This study has also provided the evidence for influence of environmental conditions on dengue virus infection. The observed average humidity and temperature during the study period indicates that less hot and humid conditions favor dengue virus infections, which might have linked with better vector growth.

The higher proportion of male victims as compared to females among the effected patients is evident in this study. Similar observations were also reported by a recent study, conducted for the analysis of dengue incidence in six Asian countries [14]. Different other studies conducted in different regions of Asia also reported the similar observations [15]–[17]. These observations indicate the gender specific difference among the dengue incidence, which might be related to exposure of dengue vector or by some unknown factor. Interestingly, the incidence of dengue infection in North America is reported either equal proportions of both females and males or females in higher proportions [18]–[20]. The phenomenon of gender specificity in relation to dengue infection might have been contributed by social, cultural (women being covered) and exposure reasons.

There is conflicting data regarding the age of affected people from dengue. Studies conducted previously in Asia, using surveillance data, correlated the age of affected patients with the severity of disease. Recent studies conducted in Pakistan suggested decrease in the median age of dengue patients during 2003–2007 and the reported age of affected individuals was 11 to 25 years [21]. Similarly, a study conducted previously in Indonesia during 1975 to 1984 reported increase in incidence of dengue infections in younger population [22]. In compliance to previous studies the median age of affected individuals was decreased from 31 years in 2011 to 21.5 years in 2012 (p<0.001), respectively. Decrease in the median age of infection suggests the development of immunity against majority of population, due to the circulation of multiple serotypes over a period of time. Additionally, severe form of disease (DHF) was reported more commonly in the younger population (Figure 2). There was increase in the frequency of DHF patients in 2012 compared with 2011; which might be contributed by infections with more virulent virus. Attack rates were highest among the age group of 31 to 45 years, while the attack rates were lowest in the younger population (till 15 years). In contrast, the children are reportedly the highly effected group by dengue virus infection [23], [24]. The high frequency of cases in the age group of 16 to 30 years in this study was due to larger population size in that age category.

Identification of diagnostic markers to predict the DHF during dengue infection is important for disease management. The clinical symptoms are helpful in diagnosing the DHF according to the WHO criteria [25]. In the current study, different clinical features and hematological abnormalities which were significantly different (p<0.05) in both DF and DHF include abdominal pain, nausea, rash, cold/clammy skin, periorbital puffiness, epistaxis, gingivitis, melena, hematemesis, hematuria, splenomegaly and hepatomegaly. While all of these features were also presented by DF patients, the clinical significance of these abnormalities in predicting the disease severity is restricted. However combination of these rare symptoms could be used as predictors of DHF [21], [25]. Periorbital puffiness, referred to swelling of the orbits due to fluid buildup around the eyes is a new observation in this study, associated significantly with DHF. However further research is required to determine the clinical significance of this sign in determining disease severity.

Most patients in this study had thrombocytopenia, leucopenia, elevated liver enzymes and hematological abnormalities. The ALT levels were up to 2.7 times higher than normal in DHF patients 74.2% (p = 0.032) while AST levels were raised up to 10 times than normal in all DHF patients; which indicates their significance in predicting the disease severity. The liver injury is very common in Dengue virus infection, which is being mediated by the infection of hepatocytes and Kupffer cells by the virus [26], [27]. Likewise the use of ALT and AST levels are suggested recently for evaluating the severity of dengue virus infection. Different studies have reported the prediction of disease severity using the AST and ALT levels [21], [28] other than that, Splenomegaly was significantly (p<0.05) higher in DHF patients. Dengue virus replicates predominantly in spleen, thymus and lymph nodes, resulting in lymphadenopathy and splenomegaly in DHF. Splenic rupture may occur as a more sever complication of splenomegaly [28]. Amazingly hematocrit, an indicator of capillary leakage was higher in DF (42.05±8.25) than DHF (36±17.8) patients, the phenomenon might be contributed by overt/occult bleed [11].

Preexisting co-morbidities in dengue infection are considered to be risk factor for the development of severe dengue fever (DHF) [29]. Moreover, higher case fatalities were significantly associated with co-morbidities [30]. The odds to develop severe dengue fever (DHF/DSS), in the present study, were significantly higher (OR 2.05, 95% CI 1.2 to 3.4, p = 0.005) in patients with co-morbidities as compared to dengue infection alone. Previously diabetes, hypertension and allergies were shown to be associated with sever manifestations of dengue fever [29], [30], [31], however there is scarcity of the data available on this topic, especially with hepatitis C virus or malarial parasite co-infection. HCV co-infection significantly contributed in the severity of the dengue infection. Odds to develop severe dengue fever was significantly higher (OR 2.1, 95% CI 1.1 to 4.1, p 0.02) in patients with HCV co-infection than in dengue infection alone. However, these results cannot be generalized due to limited sample size and further studies are warranted to confirm the outcome of dengue infection in multiple co-morbidities.

## Conclusions

Demographic, clinical and laboratory features of dengue cases studied could be used for the early diagnosis and treatment of the patients at risk of severe dengue fever. Abdominal pain, nausea, rash, cold/clammy skin, periorbital puffiness, elevated liver enzymes and visceromegaly could be used as predictors of DHF. The areas spotted in this study from which dengue cases reported more frequently, could be focused by policy makers, educationists and health professionals for the control of dengue fever in the city. Co-morbidities in dengue infection are considered to be risk factors for DHF.
