# Correction: Demographic and Clinico-Epidemiological Features of Dengue Fever in Faisalabad, Pakistan

**DOI:** 10.1371/journal.pone.0112196

**Published:** 2014-10-24

**Authors:** The PLOS ONE Staff

## Notice of Republication

This article was republished on August 4, 2014, to replace copyrighted or confidential material. Please download this article again to view the correct version.
