# Correction: Demographic and Clinico-Epidemiological Features of Dengue Fever in Faisalabad, Pakistan

**DOI:** 10.1371/journal.pone.0113174

**Published:** 2014-11-05

**Authors:** The PLOS ONE Staff

There is an error in the legend for Figure 2. Please see the complete, corrected Figure 2 here.

**Figure 2:**
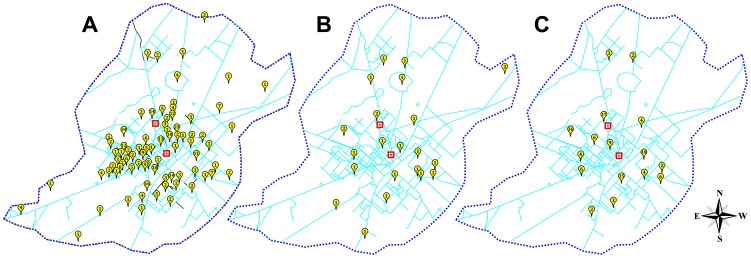
Hot spots for dengue infection in Faisalabad city. The distribution of dengue cases reported from Faisalabad city. A) Map of Faisalabad city showing areas from which dengue cases were reported in 2011, B) in 2012 and C) during both years. Squared place marks represents location of medical centers in the city.

Table 2 has been corrected for improved readability. Please see the corrected Table 2 here.

**Table 2:** Characteristics Presented by Patients Suffering from Dengue Fever (DF) and Dengue Hemorrhagic Fever (DHF).

Characteristics	DF (%)	DHF (%)	N	p value^1^
**Demographics**				
Age [median (interquartile range)]	30 (22)	24.5 (17)	299	0.05
Gender				
Male	184 (77)	34 (56.7)	299	0.03
Female	55 (23)	26 (43.3)		
**Clinical Presentation**				
Headache	184 (77)	46 (76.7)	299	>0.05
Myalgia/arthralgia	212 (88.7)	49 (81.6)	299	0.191
Vomiting	121 (50.6)	35 (58.3)	299	0.314
Abdominal pain	34 (14.2)	21 (35)	299	0.001
Nausea	36 (15.1)	17 (28.3)	299	0.023
Rash	16 (6.7)	12 (20)	299	0.005
Diarrhea	11 (4.6)	5 (8.3)	299	0.331
Anorexia	13 (5.4)	9 (15)	299	0.22
Sore throat	16 (6.7)	6 (10)	299	0.407
Retro-orbital pain	17 (7.1)	9 (15)	299	0.07
Cough	22 (9.2)	7 (11.6)	299	0.625
Cold skin/clammy skin	4 (1.7)	5 (8.3)	299	0.018
Restlessness	11 (4.6)	7 (11.6)	299	0.062
Periorbital puffiness	5 (2.1)	5 (8.3)	299	0.031
**Hemorrhages**				
Epistaxis (Nose bleed)	14 (5.9)	15 (25)	299	<0.001
Gingivitis (Gum Bleed)	17 (7.1)	11 (18.3)	299	0.012
Melena (blood in stool)	9 (3.8)	12 (20)	299	<0.001
Hematemesis (vomiting blood)	13 (5.4)	8 (13.3)	299	0.045
Hematuria (blood in urine)	5 (2.1)	5 (8.3)	299	0.031
Petechiae (small spots)	12 (5)	7 (11.7)	299	0.074
Other*	8 (3)	7 (11.7)	299	0.016
**Clinical Signs**				
Temperature [median (SD)]	37 (0.2)	37.22 (1.1)		0.108
Abdominal tenderness	7 (2.9)	8 (13.3)	299	0.003
Splenomegaly (Spleen enlargement)	15 (6.3)	14 (23.3)	299	<0.001
Hepatomegaly (Liver enlargement)	12 (5)	18 (30)	299	<0.001
**Laboratory Findings**				
Thrombocytopenia at presentation	226 (96.2)	57(95)	295	0.715
Low hemoglobin	104 (44.1)	43 (71.7)	296	<0.001
Hematocrit level on admission [median (SD)]	42.05 (8.3)	36 (17.9)	296	<0.001
Leukopenia	172 (72.3)	42 (70)	298	0.749
Increased ALT	112/213 (52.6)	23/31 (74.2)	244	0.032
Increased AST	08/14 (57.1)	05/05 (100)	19	0.077

1 p-value was calculated by non-parametric (Mann-Whitney U) test for age, temperature, and hematocrit. The χ^2^-test was used for all other categorical variables; p value less than 0.05 was considered as significant.

*Menstrual bleed, Sub-conjunctival bleed, Ear Bleed, Hemoptysis, Purpura, Ecchymosis.

The “Cluster Map for Dengue Infection” subsection of the Materials and Methods is incorrect. The correct paragraph is: CorelDRAW 12 was used to build map of the Faisalabad city in which placemarks indicate areas from which dengue cases were reported. Numbers on the placemarks represent the frequency of dengue cases from the specified areas.
